# Supplementary material for: Supramolecular Self‐Assembly as a Tool To Preserve the Electronic Purity of Perylene Diimide Chromophores
Source: Angew Chem Int Ed Engl. 2023 Feb 8;62(12):e202216729. doi: 10.1002/anie.202216729 (PMC10947190; doi:10.1002/anie.202216729)
Supplement: Supplementary file 1 — Supporting Information [file ANIE-62-0-s001.pdf]

Angewandte Chemie
Eine Zeitschrift der Gesellschaft Deutscher Chemiker

Supporting Information

Supramolecular Self-Assembly as a Tool To Preserve the Electronic Purity of Perylene Diimide Chromophores

*I. Heckelmann, Z. Lu, J. C. A. Prentice, F. Auras, T. K. Ronson, R. H. Friend, J. R. Nitschke, S. Feldmann**

Supplementary Information

Methods

Synthesis details

All reactions were performed in oven-dried glassware under argon atmosphere using standard Schlenk and glovebox techniques. Reagents and solvents were obtained in high-purity grades from commercial suppliers and were, unless shipped under argon, degassed and saturated with argon prior to use. Flash column chromatography was performed using silica gel (Acros Organics, 60 Å, 40 – 60 µm) and was continuously monitored via thin layer chromatography (TLC) using silica gel coated aluminum plates (Merck, 60 Å, F254).
Nuclear magnetic resonance (NMR) spectra were recorded on Bruker Avance III HD spectrometers. Chemical shifts are expressed in parts per million (δ scale) and are calibrated using the (undeuterated) solvent signals as an internal reference (^{1}H NMR: $CDCl_3$: 7.26, DMSO-d_6: 2.50; ^{13}C NMR: $CDCl_3$: 77.2, DMSO-d_6: 39.5). Data for ^{1}H NMR spectra are reported in the following way: chemical shift (δ ppm) (multiplicity, coupling constant, integration). Multiplicities are reported as follows: s = singlet, d = doublet, t = triplet, q = quartet, p = quintet, m = multiplet, br = broad, or combinations thereof. 13C NMR assignments were supported by DEPT-135 spectra where necessary. Low-resolution electrospray ionization (ESI) mass spectra were obtained on a Micromass Quattro LC mass spectrometer (cone voltage 10-30 eV; source temperature 313 K) infused from a Harvard Syringe Pump at a rate of 10 µL min^{-1}.

***N*,*N*'-bis(3-pentyl)perylene-3,4,9,10-bis(dicarboximide)-2,5,8,11-tetrakis(boronic acid pinacol ester) (1)**

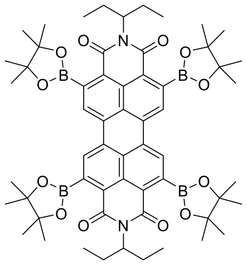

Following a published procedure,[1] a reaction mixture containing *N*,*N*'-bis(3-pentyl)perylene-3,4,9,10-bis(dicarboximide)[2] (2.65 g, 5.00 mmol, 1.0 eq.), $[Ir(OMe)(cod)]_2$ (166 mg, 0.25 mmol, 5%), $P(C_6F_5)_3$ (532 mg, 1.00 mmol, 20%), and $(Bpin)_2$ (10.2 g, 40.0 mmol, 8.0 eq.) in 240 mL anhydrous 1,4-dioxane was stirred at 110 °C for 3 d under argon. After cooling to room temperature, all volatiles were removed under reduced pressure. The solid residue was purified via column chromatography (silica gel, gradient DCM + 2% EtOAc to DCM + 6% EtOAc). All volatiles were removed under reduced pressure. The product was mixed with 12 mL cyclohexane, sonicated, collected by filtration, washed with cyclohexane (2 × 6 mL), and dried under high vacuum to yield the title compound as a bright orange powder (3.95 g, 3.82 mmol, 76%).

^{1}H NMR (500 MHz, $CDCl_3$): δ 8.52 (s, 4H), 4.91 (tt, J = 8.9, 6.2 Hz, 2H), 2.23 – 2.11 (m, 4H), 2.00 – 1.89 (m, 4H), 1.54 (s, 48H), 0.92 (t, J = 7.5 Hz, 12H).

^{13}C NMR (126 MHz, $CDCl_3$): δ 165.9, 138.6, 133.3, 128.2, 127.1, 126.9, 125.9, 84.6, 58.2, 25.2, 11.8. The C-B signal is not observed due to slow relaxation.

***N*,*N*'-bis(3-pentyl)-2,5,8,11-tetrakis(4-*N*-Boc-aminophenyl)perylene-3,4,9,10-bis(dicarboximide) (2)**

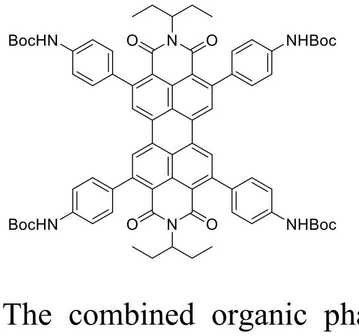

A reaction mixture containing compound **1** (1.66 g, 1.60 mmol, 1.0 eq.), *N*-(*tert*-butoxycarbonyl)-4-bromaniline (4.35 g, 16.0 mmol, 10 eq.), K_2CO_3 (1.77 g, 12.8 mmol, 8.0 eq.), $Pd_2(dba)_3 \cdot CHCl_3$ (331 mg, 0.32 mmol, 20%), and SPhos (263 mg, 0.64 mmol, 40%) in 25.6 mL *o*-xylene and 6.4 mL H_2O was stirred at 100 °C for 2 d under argon. After cooling to room temperature, the reaction mixture was mixed with brine and extracted with DCM. The combined organic phases were dried over $MgSO_4$ and concentrated under reduced pressure. The product was purified by column chromatography (silica gel, gradient DCM + 5% EtOAc to DCM + 12% EtOAc). The combined product fractions were concentrated under reduced pressure to about 15 mL, collected by filtration, washed with 15 mL EtOAc and dried under high vacuum to yield the title compound as a bright red powder (1.19 g, 0.919 mmol, 57%). This compound was used directly in the next step.

***N*,*N*'-bis(3-pentyl)-2,5,8,11-tetrakis(4-aminophenyl)perylene-3,4,9,10-bis(dicarboximide) (L1)**

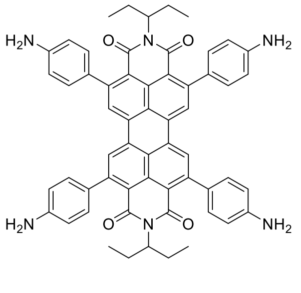

A solution of compound **2** (79 mg, 0.061 mmol, 1.0 eq.) in 9 mL anhydrous DCM and 3 mL TFA was stirred at room temperature for 2 h. After completion, the reaction mixture was neutralised by slow addition of aqueous $NaHCO_3$ and extracted with $CHCl_3$. The combined organic phases were dried over $MgSO_4$ and concentrated under reduced pressure until almost dry. MeOH (3 mL) was added and the mixture was sonicated. The precipitate was collected by

filtration and dried under high vacuum to yield the title compound as a dark purple powder (48 mg, 0.054 mmol, 88%).

^{1}H NMR (700 MHz, DMSO-d_6): δ 8.56 (s, 4H), 7.21 (d, J = 8.3 Hz, 8H), 6.64 (d, J = 8.2 Hz, 8H), 5.36 (s, 8H), 4.68 (tt, J = 10.3, 5.4 Hz, 2H), 2.08 – 1.99 (m, 4H), 1.73 – 1.64 (m, 4H), 1.17 (t, J = 7.3 Hz, 12H).

^{13}C NMR (176 MHz, DMSO-d_6): δ 164.0, 148.9, 147.6, 132.1, 130.9, 130.1, 128.8, 127.1, 123.3, 119.7, 113.5, 57.0, 24.6, 8.7.

Pseudo-cube C1

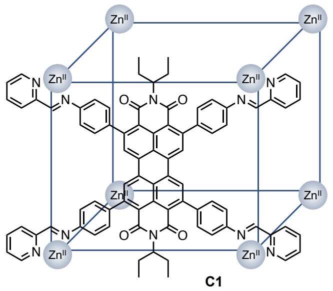

C1

Zinc bis(trifluoromethane)sulfonimide (3.00 mg, 4.8 µmol, 9 eq.), achiral PDI ligand **L1** (2.86 mg, 3.2 µmol, 6 eq.) and 2-formylpyridine (1.5 µL, 15.8 µmol, 30 eq.) were dissolved in acetonitrile (0.6 mL). The reaction mixture was stirred under nitrogen at 80 °C overnight. Diethyl ether (30 mL) was added and the resulting solid was isolated by centrifugation and washed once with fresh diethyl ether. Excess solvent was blown off with nitrogen and the residue was dried in vacuo to afford the solid product as a fine magenta powder (6.80 mg, 0.54 µmol, 98%).

The ^{1}H NMR obtained at 298 K is very broad and does not provide much information, see below.

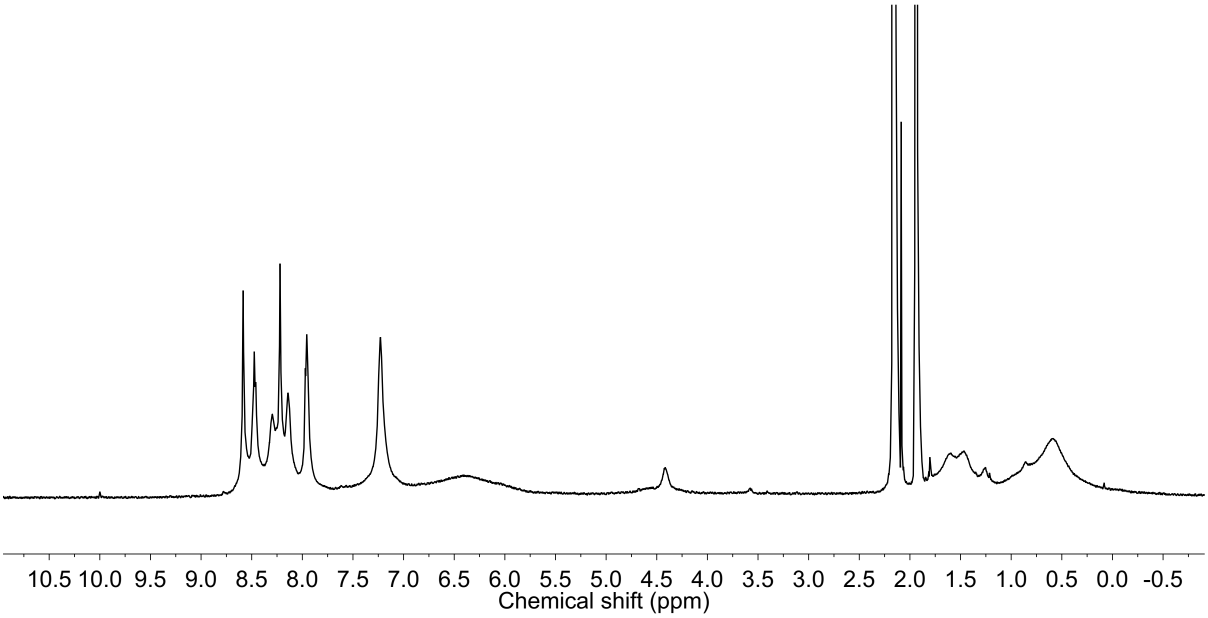

However, when using VT-NMR, a gradual sharpening of peaks was observed upon increasing the temperature. Here the data recorded at 348 K is presented:

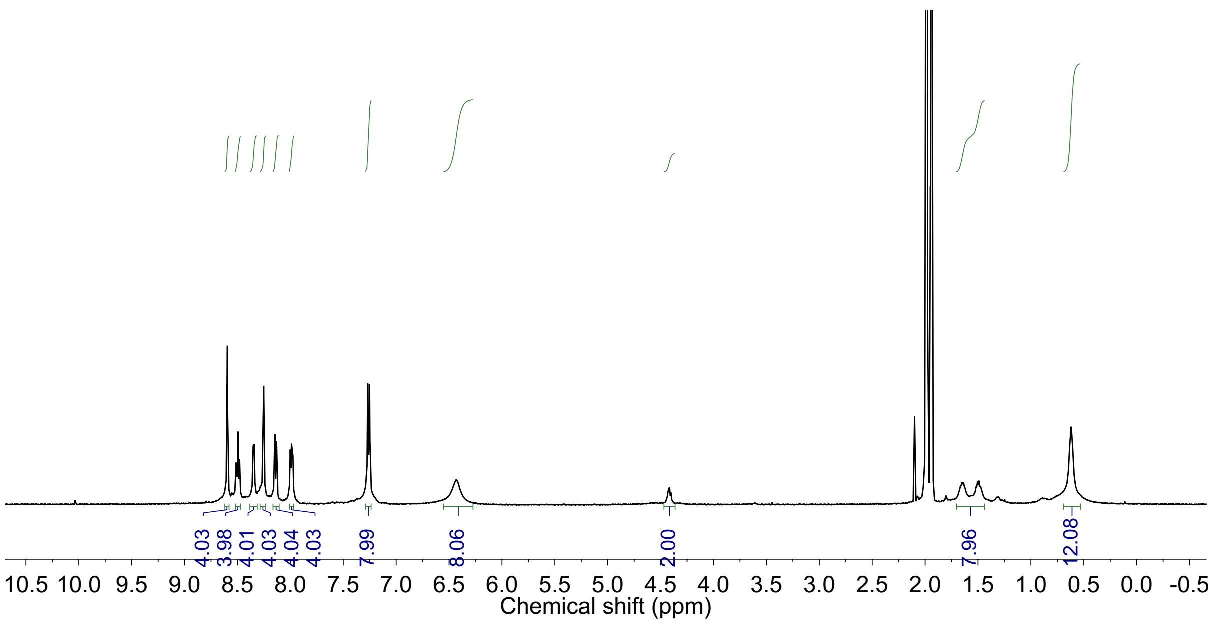

^{1}H NMR (500 MHz, CD_3CN) δ 8.60 (s, 4H), 8.47 – 8.52 (m, 4H), 8.35 (d, *J* = 4.7 Hz, 4H), 8.25 (s, 4H), 8.14 (d, *J* = 7.7 Hz, 4H), 7.97 – 8.01 (m, 4H), 7.26 (d, *J* = 8.4 Hz, 8H), 6.28 – 6.55 (m, 8H), 4.36 – 4.47 (m, 2H), 1.44 – 1.70 (m, 8H), 0.54 – 0.70 (s, 12H).
^{13}C NMR data could not be obtained due to low solubility.

ESI-MS [charge, calculated for $C_{524}H_{372}F_{96}N_{76}O_{88}S_{32}Zn_8$]:

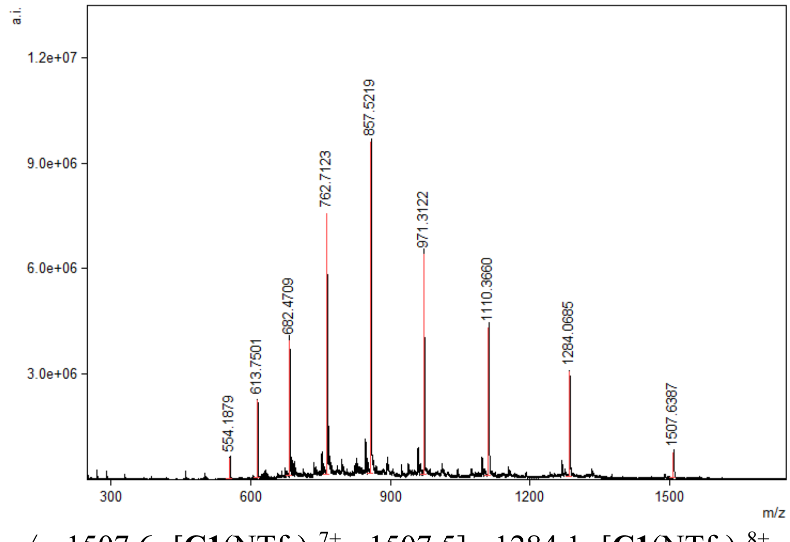

m/z 1507.6 [**C1**$(NTf_2)_9{}^{7+}$, 1507.5], 1284.1 [**C1**$(NTf_2)_8{}^{8+}$, 1284.1], 1110.4 [**C1**$(NTf_2)_7{}^{9+}$, 1110.3], 971.3 [**C1**$(NTf_2)_6{}^{10+}$, 971.2], 857.5[**C1**$(NTf_2)_5{}^{11+}$, 857.5], 762.7 [**C1**$(NTf_2)_4{}^{12+}$, 762.7], 682.5 [**C1**$(NTf_2)_3{}^{13+}$, 682.5], 613.8 [**C1**$(NTf_2)_2{}^{14+}$, 613.7], 554.2 [**C1**$(NTf_2)_1{}^{15+}$, 554.1].

X-ray crystallography

Data were collected at Beamline I19 of Diamond Light Source employing silicon double crystal monochromated synchrotron radiation (0.6889 Å) with ω and ψ scans at 100(2) K.[3] Data integration and reduction were undertaken with Xia2.[4] Subsequent computations were carried out using the WinGX-32 graphical user interface.[5] Multi-scan empirical absorption corrections were applied to the data using the AIMLESS[6] tool in the CCP4 suite[7] or DIALS.[8] The structures were solved by direct methods using SHELXT[9] then refined and extended with SHELXL.[10] In general, non-hydrogen atoms with occupancies greater than 0.5 were refined anisotropically. Carbon-bound hydrogen atoms were included in idealised positions and refined using a riding model. Oxygen-bound hydrogen atoms were first located in the difference Fourier map before refinement. Disorder was modelled using standard crystallographic methods including constraints and restraints where necessary. Crystallographic data have been deposited with the CCDC (CCDC 2215167).

The **C1** crystals with composition $[Zn_8L_6]\cdot 16\ NTf_2\cdot Et_2O$ were grown by vapour diffusion of diethyl ether into an acetonitrile solution of the complex. The crystals employed in this study proved to be weakly diffracting and rapidly suffered solvent loss. Rapid handling prior to quenching in liquid nitrogen was required to collect data. Despite these measures and the use of synchrotron radiation the diffraction pattern was broad, and the intensity of reflections dropped off rapidly past 1.0 Å and the data were trimmed accordingly. Nevertheless, the quality of the data is far more than sufficient to establish the connectivity of the structure. The asymmetric unit was found to contain one half of a Zn_6L_6 assembly plus associated solvents and counterions.

Due to the limited resolution bond lengths and angles within pairs of chemically identical organic ligands were restrained to be similar to each other and some additional bond length restraints (DFIX) were applied where necessary. One of the central PDI panels was modelled as disordered over two locations and another PDI panel showed evidence of disorder at one end. Additional bond length and angle restraints (DFIX, DANG, FLAT), generated using the GRADE program[11] was employed using the GRADE Web Server,[12] were applied to the disordered sections of the ligands. Thermal parameter restraints (SIMU, RIGU) were applied to all atoms except for zinc to facilitate anisotropic refinement.

The anions within the structure show evidence of substantial disorder. Four of the located triflimide anions molecules were modelled as disordered over two or three locations. The occupancies of these disordered anions were allowed to refine freely. Substantial bond length and thermal parameter restraints were applied to facilitate a reasonable refinement of the disordered triflimide anions and most low occupancy disordered groups were modelled with isotropic thermal parameters.

Further reflecting the solvent loss and poor diffraction properties there is a significant amount of void volume in the lattice containing smeared electron density from disordered solvent and 7.6 anions per Zn_8L_6 assembly (assigned to triflimide in the formula). Consequently the SQUEEZE[13] function of PLATON[14] was employed to remove the contribution of the electron density associated with these remaining anions and further highly disordered solvent, which gave a potential solvent accessible void of 4681 Å^3 per unit cell (a total of approximately 1544 electrons). Diffuse solvent molecules could not be assigned to acetonitrile or diethyl ether and

were therefore not included in the formula. Consequently, the molecular weight and density given above are underestimated.
CheckCIF gives a single A level alert due to the limited resolution of the data.

Identification code	zl418_sq	
Empirical formula	C528 H382 F96 N76 O89 S32 Zn8	
Formula weight	12587.95	
Temperature	100(2) K	
Wavelength	0.6889 Å	
Crystal system	Triclinic	
Space group	P -1	
Unit cell dimensions	a = 24.2902(4) Å	a= 114.1130(10)°.
	b = 27.7163(2) Å	b= 102.1480(10)°.
	c = 28.0300(4) Å	g = 101.0750(10)°.
Volume	15999.9(4) $Å^3$	
Z	1	
Density (calculated)	1.306 Mg/m^3	
Absorption coefficient	0.455 mm^{-1}	
F(000)	6410	
Crystal size	0.030 x 0.020 x 0.020 mm^3	
Theta range for data collection	0.815 to 20.148°.	
Index ranges	-24<=h<=23, -27<=k<=27, -28<=l<=28	
Reflections collected	109246	
Independent reflections	33289 [R(int) = 0.0587]	
Completeness to theta = 20.148°	99.4 %	
Absorption correction	Empirical	
Max. and min. transmission	1.0 and 0.9813351786215286	
Refinement method	Full-matrix least-squares on F^2	
Data / restraints / parameters	33289 / 9451 / 3745	
Goodness-of-fit on F^2	1.089	
Final R indices [I>2sigma(I)]	R1 = 0.1102, wR2 = 0.3130	
R indices (all data)	R1 = 0.1591, wR2 = 0.3428	
Largest diff. peak and hole	0.728 and -0.413 e.$Å^{-3}$	

Supplementary Table T1: Crystal data and structure refinement for C1.

Steady-state absorption

A Shimadzu UV-3600 Plus spectrophotometer was used to collect the steady-state absorbance spectra of samples, which uses a photomultiplier tube. The final data shown is corrected for by measuring the same cuvette with the solvent only and subtracting this spectrum from the one containing the ligand or cage, respectively.

Steady-state and time-resolved photoluminescence (PL)

Steady-state and time-resolved PL spectra were recorded by a gated intensified CCD camera (Andor Star DH740 CCI-010) connected to a grating spectrometer (Andor SR303i). The pulsed output from a mode-locked Ti:sapphire optical amplifier (Spectra-Physics Solstice, 1.55 eV photon energy, 80 fs pulse width, 1 kHz repetition rate) was used to produce 400 nm excitation via second harmonic generation in a β-barium borate crystal. The iCCD gate (width 2 ns) was electronically stepped in 2 ns increments, relative to the pump pulse, to enable ns-temporal resolution of the PL decay. Faster (~100 ps resolved) kinetics were recorded using time-correlated single-photon counting (TCSPC), employing a Picoquant system at 405 nm excitation. The instrument response function (IRF) was determined using a solution of silica (glass) colloidal spheres to detect the laser scatter under otherwise identical excitation and detection conditions. This IRF was then used for the fitting of the fast time traces through convolution of the IRF with the fitting function to accurately describe the measured data using the model described below.

Photoluminescence quantum efficiency (PLQE)

PLQE data was collected using the method described by de Mello *et al.*[15]. Briefly, samples were positioned in an integrating sphere and excited at 400 nm, while the PL was collected with an Andor Shamrock spectrometer and Andor iDus CCD array. A corrected value is then determined by collecting the light from the sphere without a sample, without hitting the sample and with hitting the sample, respectively. Stated values were determined on triplicate samples which were each measured thrice, hence reporting the average of nine measurements for each composition.

Transient absorption (TA) spectroscopy

TA is a form of pump-probe spectroscopy which measures the spectrally resolved variation in absorption by a sample under photoexcitation by a pump source. By varying the pump-probe time delay, the carrier recombination kinetics of the sample can be investigated. The third harmonic of a pulsed $Nd:YVO_4$ laser (Picolo-AOT MoPa) was used as the pump beam (~1 ns pulse width, 500 Hz repetition rate, 355 nm) for the ns regime measurements. The probe spectrum was generated using a white light quasi-continuum generated through pumping a CaF_2 window with the 800 nm fundamental of a Ti:Sapphire amplifier (Spectra-Physics Solstice). A delay generator was used to electronically vary the pump-probe delay. For the short time fs-regime, the pump beam was the second harmonic (400 nm) generated by the 800 nm fundamental passing through a β-barium borate crystal. The transmitted probe and reference pulses were recorded with an NMOS linear image sensor (Hamamatsu S8381-1024Q) and processed by a customized PCI interface from Entwicklungsbüro Stresing.

Fitting model

In order to adequately fit the transient PL & TA kinetics, a multi-state model needed to be considered that follows the diagram shown in Figure 3 of the main text (reproduced here):

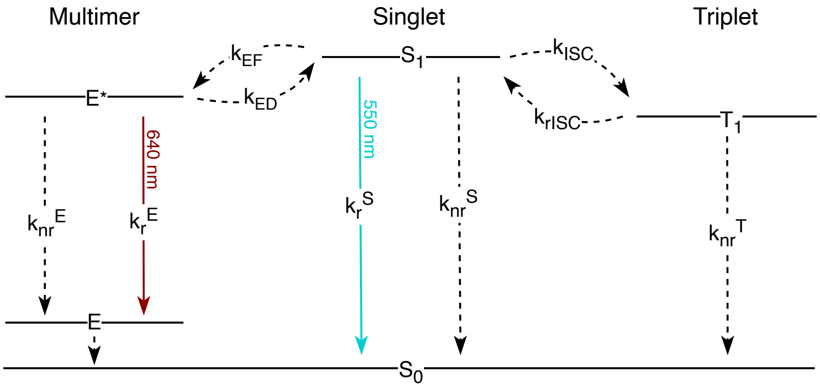

This means it was not sufficient to only consider a ground state, singlet excited state and excited multimer state, but a dark triplet state also needed to be included to describe all kinetics.
Both the (bright) excited multimer and the (dark) triplet state can act as excited state reservoir to repopulate the excited singlet state.
The time traces of the photoluminescence data were deconvoluted using non-negative matrix factorization (NMF), while the transient absorption data distinct species were separated using singular value decomposition (SVD). Then the kinetics could be fitted to a set of coupled differential equations:

$$\frac{d[S_1]}{dt} = -(k_r^S + k_{nr}^S + k_{EF} + k_{ISC})[S_1] + k_{ED}[E^*] + k_{rISC}[T_1] \quad (1)$$

$$\frac{d[E^*]}{dt} = -(k_r^E + k_{nr}^E + k_{ED})[E^*] + k_{EF}[S_1] \quad (2)$$

$$\frac{d[T_1]}{dt} = -(k_{nr}^T + k_{rISC})[T_1] + k_{ISC}[S_1] \quad (3)$$

where $[S_1]$ is the population of the excited singlet state, $[E^*]$ the excited multimer state, and $[T_1]$ the excited triplet state. k_r and k_{nr} describe the respective radiative and non-radiative rate constants for each species. As expected due to the large singlet-triplet energy gap, the reverse intersystem crossing rate k_{rISC} was found to be insignificant in magnitude and is thus not explicitly shown in the energy scheme in the main text. The fits of the data were then convoluted with the IRF measured for each setup to accurately capture the true signal rise and decay times. The extracted rate constants are listed further below.

Computational methods

All first principles calculations in this work were performed using density functional theory (DFT) and its derivative, time-dependent DFT (TDDFT), within the quantum chemistry code NWChem v7.0.2[16]. These methods provide an excellent balance between computational cost and accuracy for the molecular systems we investigate here. Calculations were performed for two systems: an isolated PDI molecule with phenylamine moieties, and dimers of the same molecule extracted from the cage. The effect of solvent was included using the COSMO implicit solvent model[17,18] used within NWChem. The parameters of the implicit solvent model were chosen to correspond to chloroform, reflecting the solvent used in the experiments. NWChem uses Gaussian basis sets to describe the electronic Kohn-Sham wavefunctions: here,

the cc-pVDZ basis set[19] was used for geometry optimizations, whilst the aug-cc-pVDZ basis set[20] was used for the rest of the single molecule calculations. The latter set additionally includes diffuse basis functions to help with the description of excited states, which are typically more delocalized. The slightly smaller, but still accurate, 6-31G* basis set (Hariharan & Pople, 1973; Rassolov, et al., 1998) was used for the dimer calculations, to reduce computational cost.

The major assumption of DFT and TDDFT is the approximation of the exact exchange-correlation functional by an approximate form, of which there are many varieties. Which functional to use is therefore an important factor in the accuracy of the computations. Standard functionals have known issues with describing charge transfer correctly, which was expected to play an important role in this system. However, previous work has shown that long-range corrected hybrid functionals are able to reproduce intramolecular charge transfer accurately, with the optimally tuned (OT) LRC-ωPBE functional[21] found to be especially effective[22]. For this reason, we used the OT-LRC-ωPBE functional in the majority of calculations in this work. Exceptions to this include the geometry optimizations (both ground state and excited state), and the computation of the non-adiabatic coupling vector, where the semi-local PBE functional was used[23] rather than OT-LRC-ωPBE. PBE was used in these situations as it reduced computational costs significantly, whilst still maintaining an acceptable degree of accuracy. For the dimer calculations, the PBE0 functional (Adamo & Barone, 1999) was used, again to reduce computational cost whilst maintaining a reasonable description of the excitations.

The range separation parameter ω in the OT-LRC-ωPBE functional must be tuned to the system in question to achieve high accuracy. This is done by minimizing the value of $J^2 = \sum_{N=Z,Z+1}[\epsilon_N^{HOMO}(\omega) - IP_N(\omega)]^2$, where ϵ_N^{HOMO} is the HOMO energy and IP_N is the ionization potential with N electrons[22]. By following this procedure, we obtained a tuned value of $\omega = 0.217$ for the PDI molecule, which is the value used throughout this work.

The calculations on the single molecule system then proceeded as follows. A ground state geometry optimization was performed on the molecule, using default tolerances, giving the DFT ground state equilibrium geometry. The average dihedral angle between the phenylamine moieties and the PDI body was calculated, and found to be 49°. The absorption spectrum, including the 10 lowest spin-permitted singlet excitations, was computed for this geometry, and the difference densities for the strongest peaks were also computed. The phenylamine ligands were then all rotated identically using the molecular viewer Avogadro[24], producing geometries with a range of other dihedral angles: 30°, 60°, 82° (the angle found in the cage), and 90°. The singlet absorption spectrum and difference densities were similarly calculated for these structures as well. These results are shown in Fig. 4a-f.

In addition, we also computed the excitation energies of the 10 lowest, formally spin-forbidden, triplet states for two dihedral angles: 49° and 82°. We also geometry-optimized the system in the excited electronic state corresponding to the strongest peak in the singlet absorption spectrum. This gave the TDDFT excited state equilibrium geometry, which had several small differences compared to the ground state equilibrium geometry, including a dihedral angle of 51° rather than 49° between the phenylamine ligands and the PDI body.

Finally, we computed the coupling between the rotation of the ligands and the electronic excitation corresponding to the strongest singlet absorption peak, by computing the non-

adiabatic coupling vector (NACR). The NACR is given by $\boldsymbol{d}_{nm}(\boldsymbol{R}) = \langle\Psi_n(\boldsymbol{r},\boldsymbol{R})|\nabla_{\boldsymbol{R}}\Psi_m(\boldsymbol{r},\boldsymbol{R})\rangle_r$, where $\Psi_n(\boldsymbol{r},\boldsymbol{R})$ is the electronic wavefunction of the nth excited state, with $\boldsymbol{r}$ and $\boldsymbol{R}$ the electronic and nuclear positions respectively[25]. This was computed numerically for each component (corresponding to the displacement of a particular atom along a particular Cartesian direction) from the time-dependent non-adiabatic coupling vector (NACT), which can be computed within NWChem's non-adiabatic molecular dynamics functionality, following ref.[25]. In turn, each atom in question was manually shifted along the direction in question by a distance of $\Delta R = 0.0005$ Å, and the NACT was computed using the excitation wavefunctions for the shifted and equilibrium geometries, with an arbitrary time-step of $\Delta t = 0.1$ a.u. (0.0024 fs). This element of the NACR was then computed by multiplying the NACT by the effective velocity $\frac{\Delta R}{\Delta t}$. The NACR was only calculated for atoms in one phenylamine ligand, and the carbon atoms constituting the central benzene ring of the PDI core. To obtain a single number for the coupling between the excitation and phenylamine rotation, the dot product of the NACR with a vector of atomic displacements corresponding to a rotation of a single phenylamine ligand was computed. This vector of atomic displacements was normalized and weighted by mass, such that $\sum_\alpha m_\alpha|\boldsymbol{u}_\alpha|^2 = 1$, where m_α and $\boldsymbol{u}_\alpha$ are the mass and vector displacement of the αth atom respectively. This result was compared against the coupling between the excitation and a breathing motion of the central benzene ring of the PDI body, calculated similarly.

Our calculations on the dimer systems proceeded as follows. From the experimental cage structure shown in Fig. 1b, two PDI molecules with phenylamine moieties corresponding to two of the faces of the cage were extracted, with hydrogen atoms added to replace bonds present in the cage structure where appropriate. Two such dimers were extracted, corresponding to the two couplings suggested in Fig. 1c. The absorption spectrum for the 10 lowest spin-permitted singlet excitations, and their corresponding difference densities, were then computed for the two dimers, making use of the Tamm-Dancoff approximation (Hirata & Head-Gordon, 1999) to reduce computational cost further whilst maintaining an accurate description of the absorption spectrum. The results are shown in Fig. S8.

Supplementary data

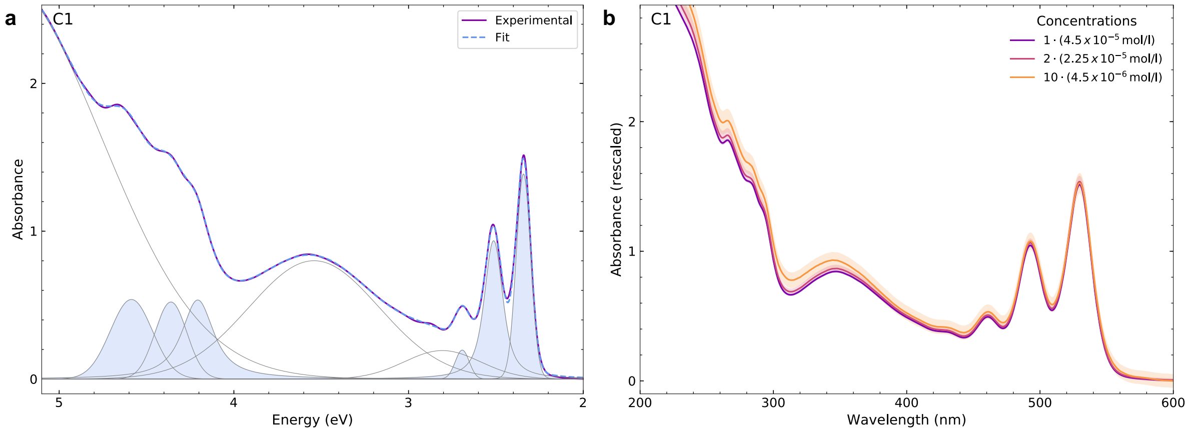

Supplementary Figure S1: UV-vis absorbance data of the supramolecular PDI cage. **a**, absorbance data and fits. **b**, Concentration series of cage absorbance showing no significant changes in absorbance features upon rescaling, in stark contrast to the aggregation effects observed for the pure PDI ligand (compare main text and Fig. 1 in there).

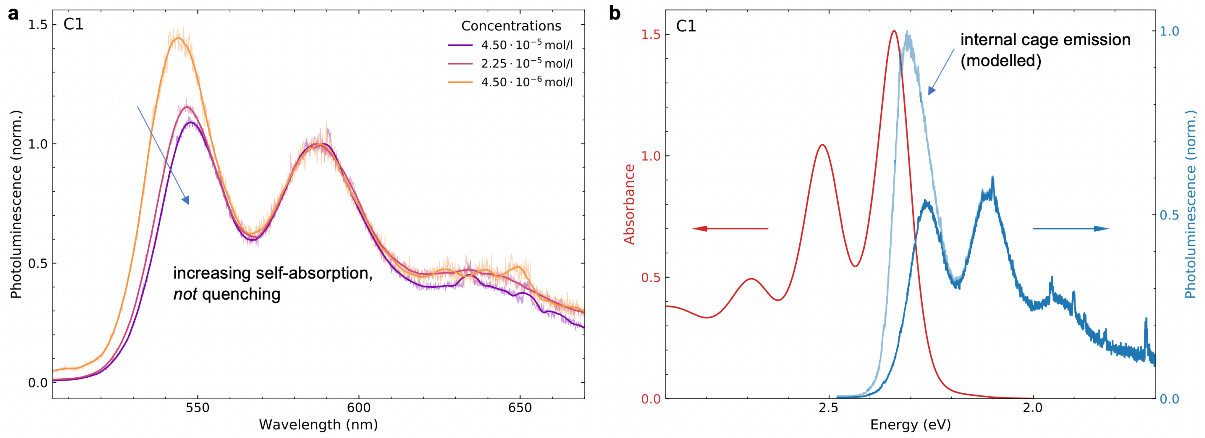

Supplementary Figure S2: Self-absorption of PDI cage explaining PL spectral feature changes at higher concentration. **a**, Concentration-dependent PL spectra showing a truncated 0-0 peak at higher concentrations for the cage. **b**, Absorbance and emission spectra for the cage at high concentration, including the modelled PL spectrum taking self-absorption into account.

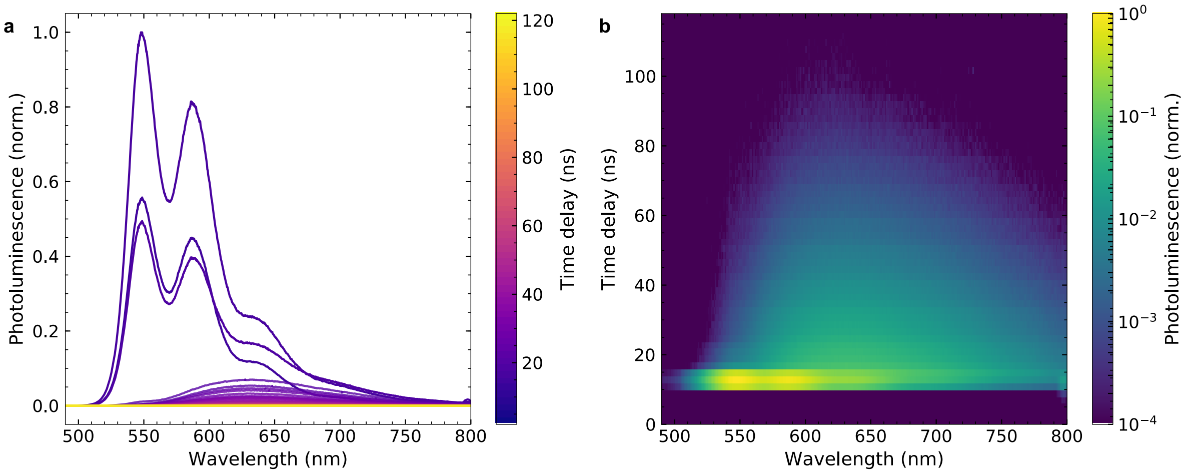

Supplementary Figure S3: Time-resolved photoluminescence data of the supramolecular PDI cage using the electrically time-gating iCCD camera. a, Individual selected time-gated spectra emphasizing the early-time prompt singlet and late-time excited multimer emission. b, 2D pseudo-color plot of the same data. The solution was excited with 400 nm pulses (ca. 100 fs pulse duration), an effective beam diameter of 363 µm, a repetition rate of 1 kHz and an average power of 22 µW.

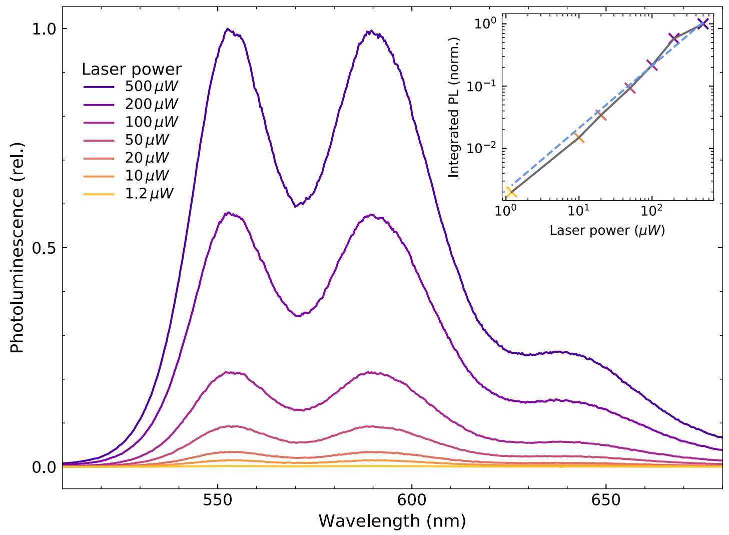

Supplementary Figure S4: Excitation fluence series of the cage emission. The emission is largely fluence-independent and scales linearly in the studied excitation density range used for all spectral measurements, as expected for excitonic recombination.

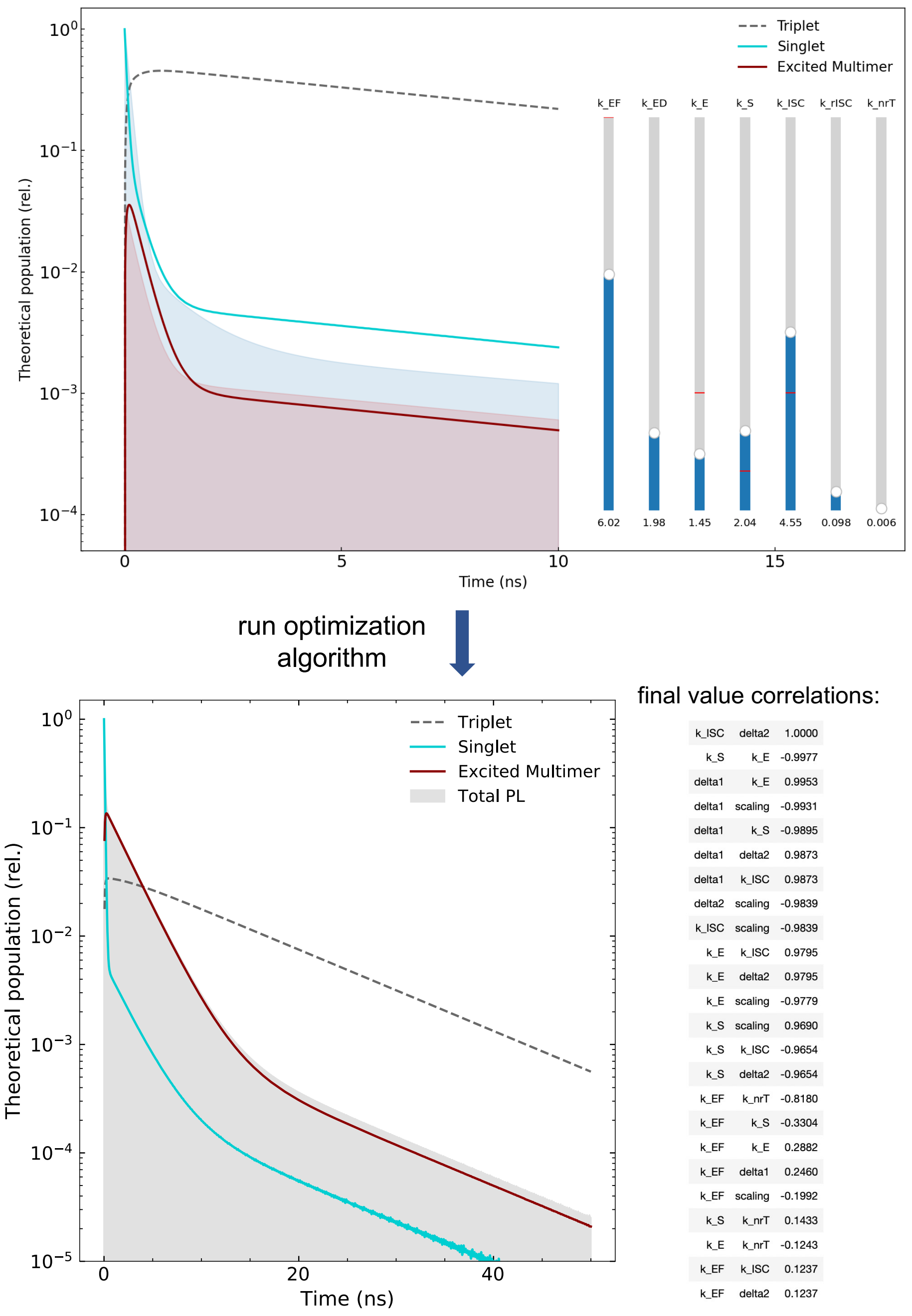

k_ISC	delta2	1.0000
k_S	k_E	-0.9977
delta1	k_E	0.9953
delta1	scaling	-0.9931
delta1	k_S	-0.9895
delta1	delta2	0.9873
delta1	k_ISC	0.9873
delta2	scaling	-0.9839
k_ISC	scaling	-0.9839
k_E	k_ISC	0.9795
k_E	delta2	0.9795
k_E	scaling	-0.9779
k_S	scaling	0.9690
k_S	k_ISC	-0.9654
k_S	delta2	-0.9654
k_EF	k_nrT	-0.8180
k_EF	k_S	-0.3304
k_EF	k_E	0.2882
k_EF	delta1	0.2460
k_EF	scaling	-0.1992
k_S	k_nrT	0.1433
k_E	k_nrT	-0.1243
k_EF	k_ISC	0.1237
k_EF	delta2	0.1237

Supplementary Figure S5: Underlying time-dependent population decay of singlet, excited multimer and triplet states, respectively, as derived from the fits to the PL data from iCCD and TCSPC measurements of the cage presented in the main text Fig. 3. The top panel shows exemplary fit parameters and populations which were then optimized to yield the final data description in the bottom panel with smallest deviation and the corresponding values in ns^{-1} (unreported correlations have values <0.100).

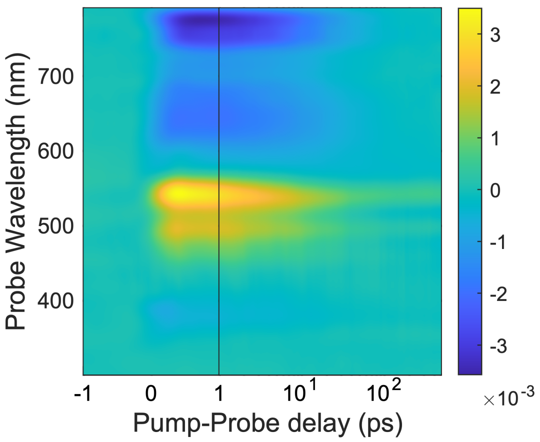

Supplementary Figure S6: 2D pseudo-color plot of the full fs-transient absorption data set of the PDI ligand.

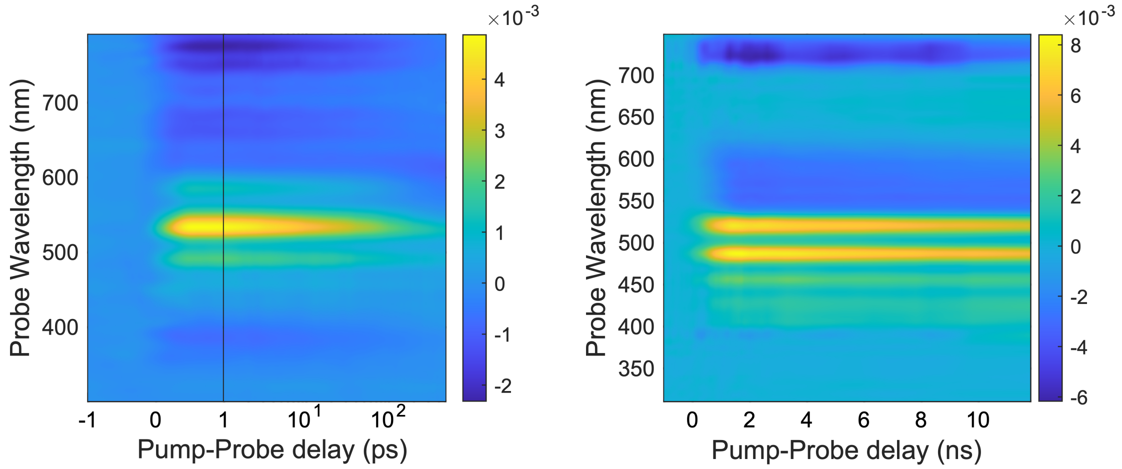

Supplementary Figure S7: 2D pseudo-color plots of the full fs- (left) and ns- (right) transient absorption data sets of the supramolecular PDI cage.

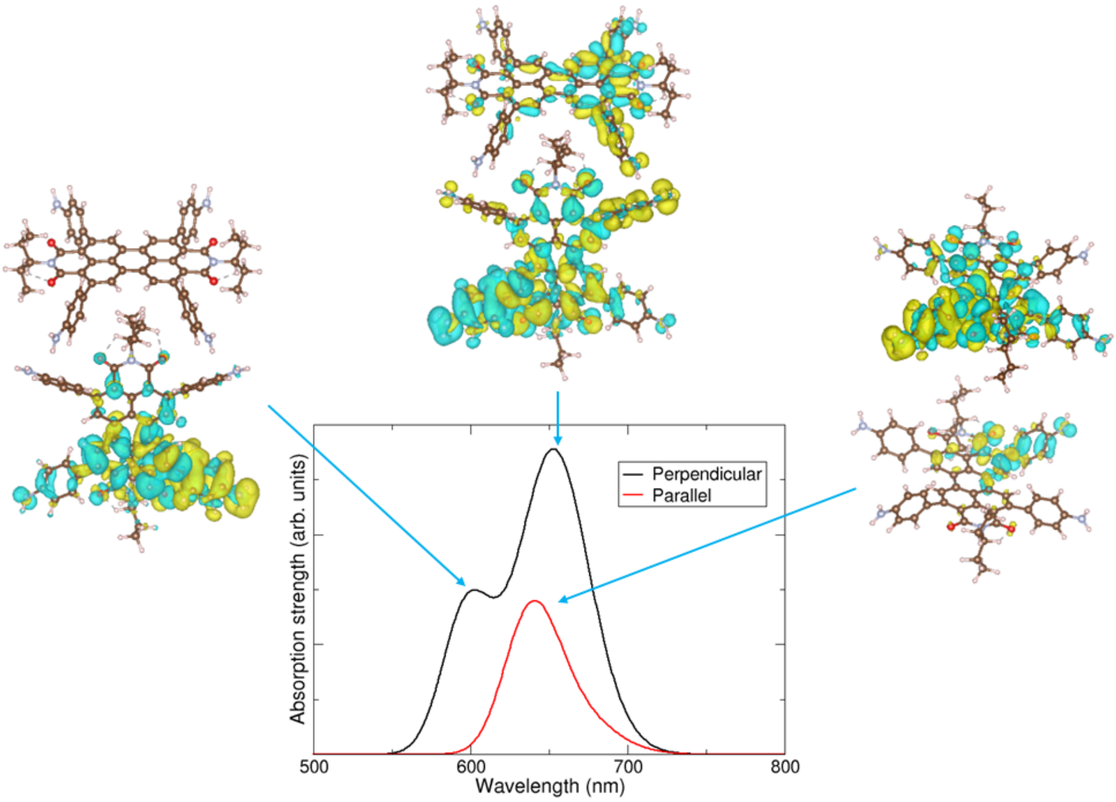

Supplementary Figure S8: Excited states difference densities and absorption spectra computed for PDI ligand dimers extracted from the cage, with both adjacent/perpendicular and opposite/parallel dimers included. Yellow represents areas of electron accumulation, and blue areas of electron depletion. Arrows indicate which peak each difference density corresponds to. Excited states localized on a single molecule (left) and delocalized across both molecules (center, right) are found. Excited states involving both molecules are at higher wavelengths, and can be seen for both perpendicular and parallel dimers.

PDI Ligand L1	rate constant (s^{-1})
k_r^S	$(2.00\pm0.04)\times10^8$
$k_{nr,tot}^S$	$(5.54\pm0.07)\times10^{10}$
k_{tot}	$(5.57\pm0.07)\times10^{10}$
k_{ISC}	$(5.0\pm1.0)\times10^{11}$
k_{nr}^S	$(3.3\pm0.6)\times10^{10}$
k_{nr}^T	$(1.5\text{-}12)\times10^{10}$
PDI Cage C1	rate constant (s^{-1})
k_{ISC}	$(5.0\pm1.0)\times10^{11}$
k_{nr}^S	$(7.7\pm0.8)\times10^9$
k_r^S	$(1.98\pm0.02)\times10^8$
k^E	$(7.81\pm0.03)\times10^7$

Supplementary Table T2: Rate constants extracted from iterative optimized fits to transient PL and TA kinetics following the fitting algorithm described above.

References

(1) Teraoka, T.; Hiroto, S.; Shinokubo, H. Iridium-Catalyzed Direct Tetraborylation of Perylene Bisimides. *Org Lett* **2011**, *13* (10), 2532–2535. https://doi.org/10.1021/ol2004534.

(2) Yang, Y.; Wang, Y.; Xie, Y.; Xiong, T.; Yuan, Z.; Zhang, Y.; Qian, S.; Xiao, Y. Fused Perylenebisimide–Carbazole: New Ladder Chromophores with Enhanced Third-Order Nonlinear Optical Activities. *Chemical Communications* **2011**, *47* (38), 10749. https://doi.org/10.1039/c1cc14071j.

(3) Allan, D. .; Nowell, H.; Barnett, S.; Warren, M.; Wilcox, A.; Christensen, J.; Saunders, L.; Peach, A.; Hooper, M.; Zaja, L.; Patel, S.; Cahill, L.; Marshall, R.; Trimnell, S.; Foster, A.; Bates, T.; Lay, S.; Williams, M.; Hathaway, P.; Winter, G.; Gerstel, M.; Wooley, R. A Novel Dual Air-Bearing Fixed-χ Diffractometer for Small-Molecule Single-Crystal X-Ray Diffraction on Beamline I19 at Diamond Light Source. *Crystals (Basel)* **2017**, *7* (11), 336. https://doi.org/10.3390/cryst7110336.

(4) Winter, G. *Xia2* : An Expert System for Macromolecular Crystallography Data Reduction. *J Appl Crystallogr* **2010**, *43* (1), 186–190. https://doi.org/10.1107/S0021889809045701.

(5) Farrugia, L. J. *WinGX* and *ORTEP for Windows* : An Update. *J Appl Crystallogr* **2012**, *45* (4), 849–854. https://doi.org/10.1107/S0021889812029111.

(6) Evans, P. R.; Murshudov, G. N. How Good Are My Data and What Is the Resolution? *Acta Crystallogr D Biol Crystallogr* **2013**, *69* (7), 1204–1214. https://doi.org/10.1107/S0907444913000061.

(7) Winn, M. D.; Ballard, C. C.; Cowtan, K. D.; Dodson, E. J.; Emsley, P.; Evans, P. R.; Keegan, R. M.; Krissinel, E. B.; Leslie, A. G. W.; McCoy, A.; McNicholas, S. J.; Murshudov, G. N.; Pannu, N. S.; Potterton, E. A.; Powell, H. R.; Read, R. J.; Vagin, A.; Wilson, K. S. Overview of the *CCP* 4 Suite and Current Developments. *Acta Crystallogr D Biol Crystallogr* **2011**, *67* (4), 235–242. https://doi.org/10.1107/S0907444910045749.

(8) Winter, G.; Waterman, D. G.; Parkhurst, J. M.; Brewster, A. S.; Gildea, R. J.; Gerstel, M.; Fuentes-Montero, L.; Vollmar, M.; Michels-Clark, T.; Young, I. D.; Sauter, N. K.; Evans, G. *DIALS* : Implementation and Evaluation of a New Integration Package. *Acta Crystallogr D Struct Biol* **2018**, *74* (2), 85–97. https://doi.org/10.1107/S2059798317017235.

(9) Sheldrick, G. M. *SHELXT* – Integrated Space-Group and Crystal-Structure Determination. *Acta Crystallogr A Found Adv* **2015**, *71* (1), 3–8. https://doi.org/10.1107/S2053273314026370.

(10) Sheldrick, G. M. Crystal Structure Refinement with *SHELXL*. *Acta Crystallogr C Struct Chem* **2015**, *71* (1), 3–8. https://doi.org/10.1107/S2053229614024218.

(11) Bricogne, G. ; B. E. ; B. M. ; F. C. ; K. P. ; P. W. ; R. P. ; S. A. ; S. O. S. ; V. C. ; W. T. O. BUSTER. 2.11.2 Ed. *Global Phasing Ltd., Cambridge, United Kingdom* **2011**.

(12) Smart, O. S.; Womack, T. O. Grade Web Server. *Global Phasing Ltd.* **2014**.

(13) van der Sluis, P.; Spek, A. L. BYPASS: An Effective Method for the Refinement of Crystal Structures Containing Disordered Solvent Regions. *Acta Crystallogr A* **1990**, *46* (3), 194–201. https://doi.org/10.1107/S0108767389011189.

(14) Spek, A. L. PLATON: A Multipurpose Crystallographic Tool. *Utrecht University, Utrecht, The Netherlands* **2008**.

(15) de Mello, J. C.; Wittmann, H. F.; Friend, R. H. An Improved Experimental Determination of External Photoluminescence Quantum Efficiency. *Advanced Materials* **1997**, *9* (3), 230–232. https://doi.org/10.1002/adma.19970090308.

(16) Aprà, E.; Bylaska, E. J.; de Jong, W. A.; Govind, N.; Kowalski, K.; Straatsma, T. P.; Valiev, M.; van Dam, H. J. J.; Alexeev, Y.; Anchell, J.; Anisimov, V.; Aquino, F. W.; Atta-Fynn, R.; Autschbach, J.; Bauman, N. P.; Becca, J. C.; Bernholdt, D. E.; Bhaskaran-Nair, K.; Bogatko, S.; Borowski, P.; Boschen, J.; Brabec, J.; Bruner, A.; Cauët, E.; Chen, Y.; Chuev, G. N.; Cramer, C. J.; Daily, J.; Deegan, M. J. O.; Dunning, T. H.; Dupuis, M.; Dyall, K. G.; Fann, G. I.; Fischer, S. A.; Fonari, A.; Früchtl, H.; Gagliardi, L.; Garza, J.; Gawande, N.; Ghosh, S.; Glaesemann, K.; Götz, A. W.; Hammond, J.; Helms, V.; Hermes, E. D.; Hirao, K.; Hirata, S.; Jacquelin, M.; Jensen, L.; Johnson, B. G.; Jónsson, H.; Kendall, R. A.; Klemm, M.;

Kobayashi, R.; Konkov, V.; Krishnamoorthy, S.; Krishnan, M.; Lin, Z.; Lins, R. D.; Littlefield, R. J.; Logsdail, A. J.; Lopata, K.; Ma, W.; Marenich, A. v.; Martin del Campo, J.; Mejia-Rodriguez, D.; Moore, J. E.; Mullin, J. M.; Nakajima, T.; Nascimento, D. R.; Nichols, J. A.; Nichols, P. J.; Nieplocha, J.; Otero-de-la-Roza, A.; Palmer, B.; Panyala, A.; Pirojsirikul, T.; Peng, B.; Peverati, R.; Pittner, J.; Pollack, L.; Richard, R. M.; Sadayappan, P.; Schatz, G. C.; Shelton, W. A.; Silverstein, D. W.; Smith, D. M. A.; Soares, T. A.; Song, D.; Swart, M.; Taylor, H. L.; Thomas, G. S.; Tipparaju, V.; Truhlar, D. G.; Tsemekhman, K.; van Voorhis, T.; Vázquez-Mayagoitia, Á.; Verma, P.; Villa, O.; Vishnu, A.; Vogiatzis, K. D.; Wang, D.; Weare, J. H.; Williamson, M. J.; Windus, T. L.; Woliński, K.; Wong, A. T.; Wu, Q.; Yang, C.; Yu, Q.; Zacharias, M.; Zhang, Z.; Zhao, Y.; Harrison, R. J. NWChem: Past, Present, and Future. *J Chem Phys* **2020**, *152* (18), 184102. https://doi.org/10.1063/5.0004997.

(17) Klamt, A.; Schüürmann, G. COSMO: A New Approach to Dielectric Screening in Solvents with Explicit Expressions for the Screening Energy and Its Gradient. *J. Chem. Soc., Perkin Trans. 2* **1993**, No. 5, 799–805. https://doi.org/10.1039/P29930000799.

(18) York, D. M.; Karplus, M. A Smooth Solvation Potential Based on the Conductor-Like Screening Model. *J Phys Chem A* **1999**, *103* (50), 11060–11079. https://doi.org/10.1021/jp992097l.

(19) Dunning, T. H. Gaussian Basis Sets for Use in Correlated Molecular Calculations. I. The Atoms Boron through Neon and Hydrogen. *J Chem Phys* **1989**, *90* (2), 1007–1023. https://doi.org/10.1063/1.456153.

(20) Kendall, R. A.; Dunning, T. H.; Harrison, R. J. Electron Affinities of the First-row Atoms Revisited. Systematic Basis Sets and Wave Functions. *J Chem Phys* **1992**, *96* (9), 6796–6806. https://doi.org/10.1063/1.462569.

(21) Vydrov, O. A.; Scuseria, G. E. Assessment of a Long-Range Corrected Hybrid Functional. *J Chem Phys* **2006**, *125* (23), 234109. https://doi.org/10.1063/1.2409292.

(22) Shee, J.; Head-Gordon, M. Predicting Excitation Energies of Twisted Intramolecular Charge-Transfer States with the Time-Dependent Density Functional Theory: Comparison with Experimental Measurements in the Gas Phase and Solvents Ranging from Hexanes to Acetonitrile. *J Chem Theory Comput* **2020**, *16* (10), 6244–6255. https://doi.org/10.1021/acs.jctc.0c00635.

(23) Perdew, J. P.; Burke, K.; Ernzerhof, M. Generalized Gradient Approximation Made Simple. *Phys Rev Lett* **1996**, *77* (18), 3865–3868. https://doi.org/10.1103/PhysRevLett.77.3865.

(24) Hanwell, M. D.; Curtis, D. E.; Lonie, D. C.; Vandermeersch, T.; Zurek, E.; Hutchison, G. R. Avogadro: An Advanced Semantic Chemical Editor, Visualization, and Analysis Platform. *J Cheminform* **2012**, *4* (1), 17. https://doi.org/10.1186/1758-2946-4-17.

(25) Song, H.; Fischer, S. A.; Zhang, Y.; Cramer, C. J.; Mukamel, S.; Govind, N.; Tretiak, S. First Principles Nonadiabatic Excited-State Molecular Dynamics in NWChem. *J Chem Theory Comput* **2020**, *16* (10), 6418–6427. https://doi.org/10.1021/acs.jctc.0c00295.
